# The vWF/ADAMTS13 Ratio as a Potential Marker of Endotheliopathy in Malignancies

**DOI:** 10.3390/biomedicines14092078

**Published:** 2026-09-15

**Authors:** Alexander Vorobev, Victoria Bitsadze, Jamilya Khizroeva, Antonina Solopova, Maria Tretyakova, Nilufar Gashimova, Kristina Grigoreva, Irina Kalashnikova, Natalia Makatsariya, Vlada Rubashkina, Yana Sulina, Aidanhanum Iskenderova, Alexandra Antonova, Dmitry Utkin, Jean-Christophe Gris, Ismail Elalamy, Grigoris Gerotziafas, Alexander Makatsariya

**Affiliations:** 1 Department of Obstetrics, Gynecology and Perinatal Medicine, I.M. Sechenov First Moscow State Medical University (Sechenov University), 8 bldg. 2, Trubetskaya Str., Moscow 119048, Russiagrigorios.gerotziafas@inserm.fr (G.G.);; 2 Moscow Healthcare Department, Yudin City Clinical Hospital, 4 Kolomensky Proezd, Moscow 115446, Russia; 3 Faculty of Pharmaceutical and Biological Sciences, University of Montpellier, 163 Rue Auguste Broussonnet, 34090 Montpellier, France; 4 Department Hematology and Thrombosis Center, Medicine Sorbonne University, 12 Rue de l’École de Médecine, 75006 Paris, France; 5 Thrombosis Center, Tenon—Saint Antoine University Hospital, 4 Rue de la Chine, 75020 Paris, France

**Keywords:** cancer-associated thrombosis, endotheliopathy, thromboinflammation, von Willebrand factor, vWF, ADAMTS13, vWF/ADAMTS13 ratio, gynecologic malignancies, chemotherapy, low-molecular-weight heparin, thromboprophylaxis

## Abstract

**Background:** Endothelial activation and thromboinflammation play an important role in cancer-associated thrombosis. An imbalance between von Willebrand factor (vWF) and its physiological regulator ADAMTS13 may reflect the development of a prothrombotic endothelial state in patients with malignancies. However, the dynamics of the vWF-ADAMTS13 axis during chemotherapy and thromboprophylaxis remain insufficiently characterized. **Objectives:** To characterize the vWF-ADAMTS13 axis as a potential laboratory marker of endotheliopathy in patients with gynecologic malignancies and to assess its dynamics during chemotherapy and thromboprophylaxis with low-molecular-weight heparin (LMWH). **Materials and Methods:** This prospective comparative study included 74 patients with stage I–III gynecologic malignancies undergoing chemotherapy after surgical treatment. Group I comprised 34 patients with a history of thrombotic complications (VTE), including 23 patients with ovarian cancer and 11 with cervical adenocarcinoma. Group II included 40 patients without previous VTE, including 20 patients with ovarian cancer and 20 with cervical adenocarcinoma. The control group consisted of 25 healthy women. Plasma vWF levels, ADAMTS13 antigen and functional activity, ADAMTS13 inhibitor levels, and the vWF/ADAMTS13 ratio were assessed before chemotherapy and after 1–2 chemotherapy cycles. Patients in Group I additionally received thromboprophylaxis with nadroparin calcium. **Results:** Before chemotherapy, an imbalance of the vWF-ADAMTS13 axis was observed in patients with malignancies compared with healthy controls and was most pronounced in patients with a history of thrombosis. The vWF/ADAMTS13 ratio was 0.65 in controls, 1.02 and 0.84 in patients without previous VTE with ovarian and cervical cancer, respectively, and 1.59 and 1.34 in the corresponding subgroups with a history of thrombosis (*p* < 0.05). After 1–2 chemotherapy cycles, the vWF–ADAMTS13 axis showed a further shift toward imbalance, characterized by increased vWF levels, decreased ADAMTS13 antigen levels and functional activity, and increased ADAMTS13 inhibitor concentrations. The vWF/ADAMTS13 ratio increased to 2.04 in patients with ovarian cancer and to 1.65 in those with cervical cancer and a history of thrombosis. In Group I, the subsequent assessment following chemotherapy and LMWH thromboprophylaxis demonstrated partial reversal of these changes: ADAMTS13 antigen levels increased, while vWF levels and ADAMTS13 inhibitor concentrations decreased, and the vWF/ADAMTS13 ratio declined from 2.04 to 1.30 in ovarian cancer and from 1.65 to 1.14 in cervical cancer. **Conclusions:** The vWF-ADAMTS13 axis is markedly altered in patients with gynecologic malignancies, including those without previous VTE, with the most pronounced imbalance observed in patients with a history of thrombosis. The post-chemotherapy assessment demonstrated a further shift toward imbalance, whereas the subsequent assessment following chemotherapy and LMWH thromboprophylaxis in Group I showed partial reversal of these changes. The vWF/ADAMTS13 ratio may represent a potential integrative and dynamic marker of endothelial–hemostatic imbalance in patients with gynecologic malignancies.

## 1. Introduction

Venous thromboembolism (VTE) is one of the most clinically significant complications of malignancy and is associated with an adverse prognosis. The pathogenesis of cancer-associated thrombosis extends beyond activation of the coagulation cascade and involves complex interactions among tumor cells, platelets, leukocytes, and the vascular endothelium. Within the current concept of thromboinflammation, endothelial activation is regarded as a key contributor to the development of a prothrombotic state in patients with malignancy, which may be further exacerbated by anticancer therapy [1].

One of the systems reflecting endothelial activation and its relationship with thrombogenesis is the von Willebrand factor (vWF)-ADAMTS13 axis. vWF is released from the Weibel–Palade bodies of activated endothelial cells and mediates platelet adhesion and aggregation, whereas the metalloprotease ADAMTS13 limits its prothrombotic activity by cleaving ultra-large and high-molecular-weight vWF multimers. In malignancy, increased vWF levels combined with a relative reduction in ADAMTS13 activity may promote a prothrombotic endothelial–hemostatic imbalance [2].

In this context, the vWF/ADAMTS13 ratio is of particular interest, as it simultaneously reflects the release of vWF from activated endothelium and the capacity of ADAMTS13 to limit its thrombogenic potential. In the prospective Vienna Cancer and Thrombosis Study, elevated vWF levels and a reduced ADAMTS13/vWF ratio were associated with subsequent VTE, whereas ADAMTS13 levels alone showed no such association [3]. In another study, an increased vWF/ADAMTS13 ratio in patients with cancer was associated with VTE and an adverse prognosis; moreover, intravascular vWF structures formed as a consequence of endothelial activation and capable of mediating platelet binding and aggregation were identified within tumor tissue [4]. These findings support the concept that an imbalance of the vWF-ADAMTS13 axis may serve as a potential integrative marker of endotheliopathy and the prothrombotic state in malignancy.

Dynamic assessment of this axis during anticancer treatment may provide additional information on treatment-associated changes in endothelial–hemostatic balance. Chemotherapy, including platinum-based agents, can induce endothelial injury and activation, thereby further promoting a prothrombotic state [5]. However, changes in the vWF-ADAMTS13 axis during chemotherapy and thromboprophylaxis remain insufficiently characterized. The potential effects of low-molecular-weight heparins (LMWH) on this system are also of interest, since, in addition to their anticoagulant activity, heparins have been reported to exert anti-inflammatory and endothelial effects, although their clinical significance has not yet been fully established [6].

In our previous study, the vWF/ADAMTS13 ratio was evaluated in relation to thrombotic risk and subsequent VTE in patients with gynecologic malignancies, with particular emphasis on its potential prognostic value [7]. The present study extends the analysis of this cohort by focusing on a distinct mechanistic question: the dynamic behavior of the vWF–ADAMTS13 axis as a marker of endothelial–hemostatic imbalance during chemotherapy and LMWH thromboprophylaxis. In addition to the vWF/ADAMTS13 ratio, the present analysis incorporates ADAMTS13 antigen levels, functional activity, and inhibitor concentrations to provide a more comprehensive assessment of this axis.

The aim of this study was to characterize the dynamic behavior of the vWF-ADAMTS13 axis as a potential laboratory marker of endotheliopathy in patients with gynecologic malignancies and to investigate changes in vWF levels, ADAMTS13 antigen levels and functional activity, ADAMTS13 inhibitor concentrations, and the vWF/ADAMTS13 ratio during chemotherapy and LMWH thromboprophylaxis.

## 2. Materials and Methods

This prospective comparative study represents an extended mechanistic analysis of a previously reported patient cohort [7]. Whereas the previous analysis primarily addressed the prognostic value of the vWF/ADAMTS13 ratio for thrombotic risk, the present study focused on serial changes in the vWF-ADAMTS13 axis as a marker of endothelial–hemostatic imbalance during chemotherapy and LMWH thromboprophylaxis, including assessment of ADAMTS13 antigen levels, functional activity, and inhibitor concentrations.

The study included 74 patients with stage I–III gynecologic malignancies who were scheduled to receive combination chemotherapy following surgical treatment. According to the presence or absence of a history of thrombotic complications, the patients were divided into two groups. Group I comprised 34 patients with a history of VTE: 23 patients with ovarian cancer (subgroup IA) and 11 patients with adenocarcinoma of the cervical canal (subgroup IB). Group II comprised 40 patients without previous VTE: 20 patients with ovarian cancer (subgroup IIA) and 20 patients with adenocarcinoma of the cervical canal (subgroup IIB). Group II was selected to provide clinical comparability with Group I with respect to age, tumor stage, and the extent and type of surgical treatment. All patients in Groups I and II were subsequently scheduled to receive postoperative chemotherapy. Detailed baseline clinical characteristics and planned chemotherapy exposure are summarized in Table S1. The control group consisted of 25 apparently healthy women without malignancies or disorders of the hemostatic system.

Exclusion criteria included severe general medical condition, severe thrombocytopenia with a platelet count <50 × 10^9^/L, signs of active bleeding, septic complications, disease progression requiring modification of anticancer therapy, and inability to undergo chemotherapy or complete serial laboratory assessment.

Laboratory assessments were performed sequentially at predefined treatment-related time points. The first assessment was performed after surgical treatment, 1–3 days before the initiation of postoperative chemotherapy. Chemotherapy was initiated after adequate postoperative recovery in accordance with standard treatment schedules for the respective malignancy; postoperative chemotherapy is generally initiated within 4–6 weeks after surgery. Thus, the first laboratory assessment represented a late postoperative, pre-chemotherapy baseline measurement rather than an assessment during the immediate postoperative period.

All patients in Groups I and II received postoperative combination chemotherapy according to tumor type, disease stage, and current clinical recommendations. The planned treatment consisted of 4–8 chemotherapy cycles. First-line treatment included platinum-based agents (cisplatin or carboplatin), paclitaxel, gemcitabine, and other cytotoxic agents indicated for the respective gynecologic malignancy. The parameters of the vWF-ADAMTS13 axis were reassessed after 1–2 chemotherapy cycles. At this time point, plasma vWF levels, ADAMTS13 antigen levels and functional activity, ADAMTS13 inhibitor concentrations, and the vWF/ADAMTS13 ratio were measured using the same laboratory methods as at the pre-chemotherapy assessment.

As previously reported for this cohort [7], patients in Group I had a history of VTE and were considered to be at high risk of recurrent thrombotic complications. Accordingly, LMWH thromboprophylaxis was administered to these patients on clinical grounds and was not assigned as a study intervention. Patients in Group II, who had no history of VTE, did not receive LMWH thromboprophylaxis. Nadroparin calcium was administered subcutaneously at a dose of 3800 anti-Xa IU once daily in patients weighing <70 kg and 5700 anti-Xa IU once daily in those weighing >70 kg. Thromboprophylaxis was initiated 6 h before the subsequent chemotherapy cycle, continued throughout the cycle, and discontinued 72 h after completion of cytotoxic drug administration. Follow-up laboratory assessment was performed 2–3 days after completion of the chemotherapy cycle and the final LMWH injection. At this assessment, plasma vWF levels, ADAMTS13 antigen levels and functional activity, ADAMTS13 inhibitor concentrations, and the vWF/ADAMTS13 ratio were reassessed; D-dimer levels were additionally determined. No corresponding time-matched third laboratory assessment was performed in Group II.

The primary laboratory parameters included ADAMTS13 antigen levels and functional activity, ADAMTS13 inhibitor concentration, vWF levels, and the calculated vWF/ADAMTS13 ratio. ADAMTS13 antigen levels and functional activity were measured using the commercially available TECHNOZYM^®^ ADAMTS13 Activity/Antigen ELISA assay (Technoclone, Vienna, Austria). ADAMTS13 inhibitor concentrations were quantitatively determined using TECHNOZYM^®^ technology. vWF levels were measured by enzyme-linked immunosorbent assay. Based on the obtained values, the vWF/ADAMTS13 ratio was calculated as an integrative parameter reflecting the balance between circulating vWF and its physiological regulator ADAMTS13.

Statistical analyses were performed using Microsoft Excel 2016 and Statistica 7.0. The distribution of quantitative variables was assessed using the Shapiro–Wilk test. Quantitative data were presented as mean ± standard deviation (M ± SD) or as the mean with minimum and maximum values [M (min–max)].

For comparisons between two independent groups, Student’s *t*-test was used for normally distributed variables, whereas the Mann–Whitney U test was applied to variables with non-normal distributions. For longitudinal within-group comparisons, paired Student’s *t*-test was used for normally distributed variables and the Wilcoxon signed-rank test for non-normally distributed variables. All statistical tests were two-sided, and *p* < 0.05 was considered statistically significant. Exact *p*-values for the comparisons presented in Table 1 and Table 2 are provided separately in Table 3 and Table 4, whereas exact *p*-values for longitudinal comparisons are reported directly in Table 5.

## 3. Results

The clinical characteristics of the study cohort are summarized in Table S1. The age distribution was similar across Groups I and II, with the majority of patients being 45–55 years of age. Tumor-stage distributions were also comparable between the corresponding subgroups. Among patients with ovarian cancer, stage III disease was present in 47.8% of subgroup IA and 45.0% of subgroup IIA; among patients with cervical adenocarcinoma, the corresponding proportions were 45.5% and 40.0% in subgroups IB and IIB, respectively. The planned number of chemotherapy cycles ranged from 4 to 8 and showed a similar distribution within the corresponding tumor-specific subgroups of Groups I and II.

After surgical treatment but before the initiation of chemotherapy, alterations in the vWF-ADAMTS13 axis were identified in patients with malignancies of the female reproductive system compared with the control group. The most pronounced abnormalities were observed in Group I, particularly among patients with ovarian cancer (subgroup IA) (Table 1).

The ADAMTS13 antigen level in subgroup IA was 1188 (803–1650) IU/L, compared with 1402 (1201–1824) IU/L in subgroup IIA and 1572 (1397–2239) IU/L in the control group. The corresponding values were 1317 (1012–1723) IU/L in subgroup IB and 1511 (1257–2032) IU/L in subgroup IIB. ADAMTS13 levels were significantly lower in subgroup IA than in subgroup IIA (*p* = 0.008) and in subgroup IB than in subgroup IIB (*p* = 0.031). Compared with the control group, ADAMTS13 levels were significantly lower in subgroups IA (*p* = 0.002), IB (*p* = 0.018), and IIA (*p* = 0.039), whereas the difference for subgroup IIB was not statistically significant (*p* = 0.341).

ADAMTS13 functional activity was lowest in subgroup IA (0.815 ± 0.256), followed by subgroup IIA (0.908 ± 0.179), subgroup IB (0.992 ± 0.187), and subgroup IIB (1.126 ± 0.185), compared with 1.256 ± 0.137 in the control group. However, differences between the corresponding subgroups with and without previous VTE did not reach statistical significance for either ovarian cancer (IA vs. IIA, *p* = 0.171) or cervical cancer (IB vs. IIB, *p* = 0.069). Compared with the control group, ADAMTS13 activity was significantly lower in subgroups IA (*p* = 0.002), IB (*p* = 0.006), and IIA (*p* = 0.003), whereas the difference in subgroup IIB was not statistically significant (*p* = 0.063).

ADAMTS13 inhibitor concentrations were markedly elevated in patients with malignancies. The highest concentrations were observed in subgroups IA (6.35 ± 2.76 IU/mL) and IB (4.91 ± 1.78 IU/mL), whereas lower values were observed in subgroups IIA (2.14 ± 0.63 IU/mL) and IIB (1.62 ± 0.78 IU/mL), compared with 0.25 ± 0.17 IU/mL in the control group. Concentrations were significantly higher in subgroup IA than in subgroup IIA (*p* = 0.001) and in subgroup IB than in subgroup IIB (*p* = 0.003). All oncologic subgroups also had significantly higher ADAMTS13 inhibitor concentrations than the control group (all *p* ≤ 0.002).

The most pronounced between-group differences were observed for vWF. The vWF level was 1892 (1239–2369) IU/L in subgroup IA and 1428 (902–1681) IU/L in subgroup IIA (*p* = 0.003), whereas the corresponding values were 1763 (1013–2092) IU/L in subgroup IB and 1272 (1087–1648) IU/L in subgroup IIB (*p* = 0.011). Compared with the control group [1014 (726–1486) IU/L], vWF levels were significantly higher in all oncologic subgroups (*p* = 0.001, *p* = 0.002, *p* = 0.006, and *p* = 0.018 for IA, IB, IIA, and IIB, respectively).

The opposing changes in vWF and ADAMTS13 were reflected in higher vWF/ADAMTS13 ratios across the oncologic subgroups. The ratio was 0.65 in the control group, 1.02 in subgroup IIA, 0.84 in subgroup IIB, 1.59 in subgroup IA, and 1.34 in subgroup IB. The ratio was significantly higher in subgroup IA than in subgroup IIA (*p* = 0.006) and in subgroup IB than in subgroup IIB (*p* = 0.009). Compared with the control group, the vWF/ADAMTS13 ratio was significantly increased in all oncologic subgroups (IA, *p* = 0.001; IB, *p* = 0.002; IIA, *p* = 0.012; IIB, *p* = 0.027).

Thus, an imbalance in the vWF-ADAMTS13 axis was observed not only in patients with a history of thrombotic complications but also in subgroups IIA and IIB, in whom no previous VTE had been documented. The most pronounced alterations were observed in subgroup IA.

After 1–2 cycles of chemotherapy, the vWF–ADAMTS13 axis showed a further shift toward imbalance across all oncologic subgroups, characterized by lower ADAMTS13 antigen levels and functional activity, higher ADAMTS13 inhibitor concentrations, and higher vWF levels compared with the pre-chemotherapy assessment. The most pronounced abnormalities after chemotherapy were observed in Group I (Table 2).

In subgroup IA, ADAMTS13 antigen levels decreased from 1188 to 943 (756–1532) IU/L and functional activity from 0.815 ± 0.256 to 0.711 ± 0.352, whereas ADAMTS13 inhibitor concentrations increased from 6.35 ± 2.76 to 7.58 ± 3.52 IU/mL. In subgroup IB, ADAMTS13 antigen levels decreased from 1317 to 1084 (813–1627) IU/L and functional activity from 0.992 ± 0.187 to 0.773 ± 0.310, whereas inhibitor concentrations increased from 4.91 ± 1.78 to 5.18 ± 2.71 IU/mL. At the post-chemotherapy assessment, ADAMTS13 antigen levels were significantly lower in Group I than in the corresponding Group II subgroups (IA vs. IIA, *p* = 0.004; IB vs. IIB, *p* = 0.018). The difference in ADAMTS13 functional activity was significant for IB versus IIB (*p* = 0.012), but not for IA versus IIA (*p* = 0.064). ADAMTS13 inhibitor concentrations were significantly higher in Group I than in the corresponding Group II subgroups (IA vs. IIA, *p* = 0.001; IB vs. IIB, *p* = 0.004).

In Group II, changes from the pre-chemotherapy assessment followed the same direction but were generally less pronounced. After chemotherapy, ADAMTS13 antigen levels were 1337 (1154–1761) IU/L in subgroup IIA and 1371 (1265–1803) IU/L in subgroup IIB, while functional activity was 0.876 ± 0.204 and 1.106 ± 0.197, respectively. ADAMTS13 inhibitor concentrations were 2.64 ± 1.04 IU/mL in subgroup IIA and 2.13 ± 0.89 IU/mL in subgroup IIB. Compared with the control group, ADAMTS13 antigen levels, functional activity, and inhibitor concentrations differed significantly from the control group in both Group II subgroups (all *p* < 0.05; Table 4).

A concurrent increase in vWF levels was observed. After chemotherapy, vWF levels were 1923 (1321–2517) IU/L in subgroup IA, 1791 (1178–2259) IU/L in subgroup IB, 1513 (1126–2141) IU/L in subgroup IIA, and 1396 (1027–2064) IU/L in subgroup IIB. At this assessment, vWF levels were significantly higher in subgroup IA than in subgroup IIA (*p* = 0.006) and in subgroup IB than in subgroup IIB (*p* = 0.021), and remained significantly higher than in the control group in all oncologic subgroups (all *p* ≤ 0.016).

The most consistent directional change was observed for the vWF/ADAMTS13 ratio. Compared with the pre-chemotherapy assessment, the ratio increased from 1.59 to 2.04 in subgroup IA, from 1.34 to 1.65 in subgroup IB, from 1.02 to 1.13 in subgroup IIA, and from 0.84 to 1.02 in subgroup IIB. At the post-chemotherapy assessment, the ratio was significantly higher in subgroup IA than in subgroup IIA (*p* = 0.006) and in subgroup IB than in subgroup IIB (*p* = 0.021). All oncologic subgroups also had significantly higher ratios than the control group (IA, *p* = 0.001; IB, *p* = 0.003; IIA, *p* = 0.009; IIB, *p* = 0.014).

Thus, the post-chemotherapy assessment demonstrated a further shift in the vWF-ADAMTS13 axis toward an endothelial–hemostatic imbalance in both patients with a history of thrombotic complications and Group II patients without previous VTE. The greatest numerical increase in the vWF/ADAMTS13 ratio was observed in subgroup IA.

In Group I, the laboratory assessment following chemotherapy and LMWH thromboprophylaxis demonstrated changes in the major components of the vWF-ADAMTS13 axis in the direction opposite to that observed after the initial chemotherapy cycles (Table 5).

In subgroup IA, ADAMTS13 antigen levels increased from 943 to 1325 IU/L (*p* = 0.004), while in subgroup IB they increased from 1084 to 1426 IU/L (*p* = 0.007). ADAMTS13 functional activity increased from 0.711 to 0.843 in subgroup IA and from 0.773 to 0.952 in subgroup IB; however, these changes did not reach statistical significance (*p* = 0.100 and *p* = 0.120, respectively).

The most pronounced changes were observed in ADAMTS13 inhibitor concentrations, which decreased from 7.58 ± 3.52 to 1.73 ± 0.57 IU/mL in subgroup IA (*p* = 0.003) and from 5.18 ± 2.71 to 1.48 ± 0.51 IU/mL in subgroup IB (*p* = 0.006).

Concurrently, vWF levels decreased from 1923 to 1684 IU/L in subgroup IA (*p* = 0.012) and from 1791 to 1567 IU/L in subgroup IB (*p* = 0.038). The vWF/ADAMTS13 ratio decreased from 2.04 to 1.30 in subgroup IA (*p* = 0.004) and from 1.65 to 1.14 in subgroup IB (*p* = 0.009).

D-dimer levels also decreased markedly, from 2783 ± 953 to 512 ± 73 ng/mL in subgroup IA (*p* = 0.001) and from 2613 ± 1021 to 497 ± 98 ng/mL in subgroup IB (*p* = 0.002).

Thus, in Group I, the assessment following chemotherapy and LMWH thromboprophylaxis demonstrated a partial reversal of the preceding alterations in the vWF-ADAMTS13 axis, including increased ADAMTS13 antigen levels, decreased ADAMTS13 inhibitor and vWF levels, and a reduction in the vWF/ADAMTS13 ratio. However, these findings should be interpreted as longitudinal changes associated with the treatment period rather than as evidence of an independent effect of LMWH, because no corresponding time-matched assessment was performed in Group II. The values nevertheless remained above those observed in the control group, indicating persistent endothelial–hemostatic imbalance.

## 4. Discussion

**Figure 1:**
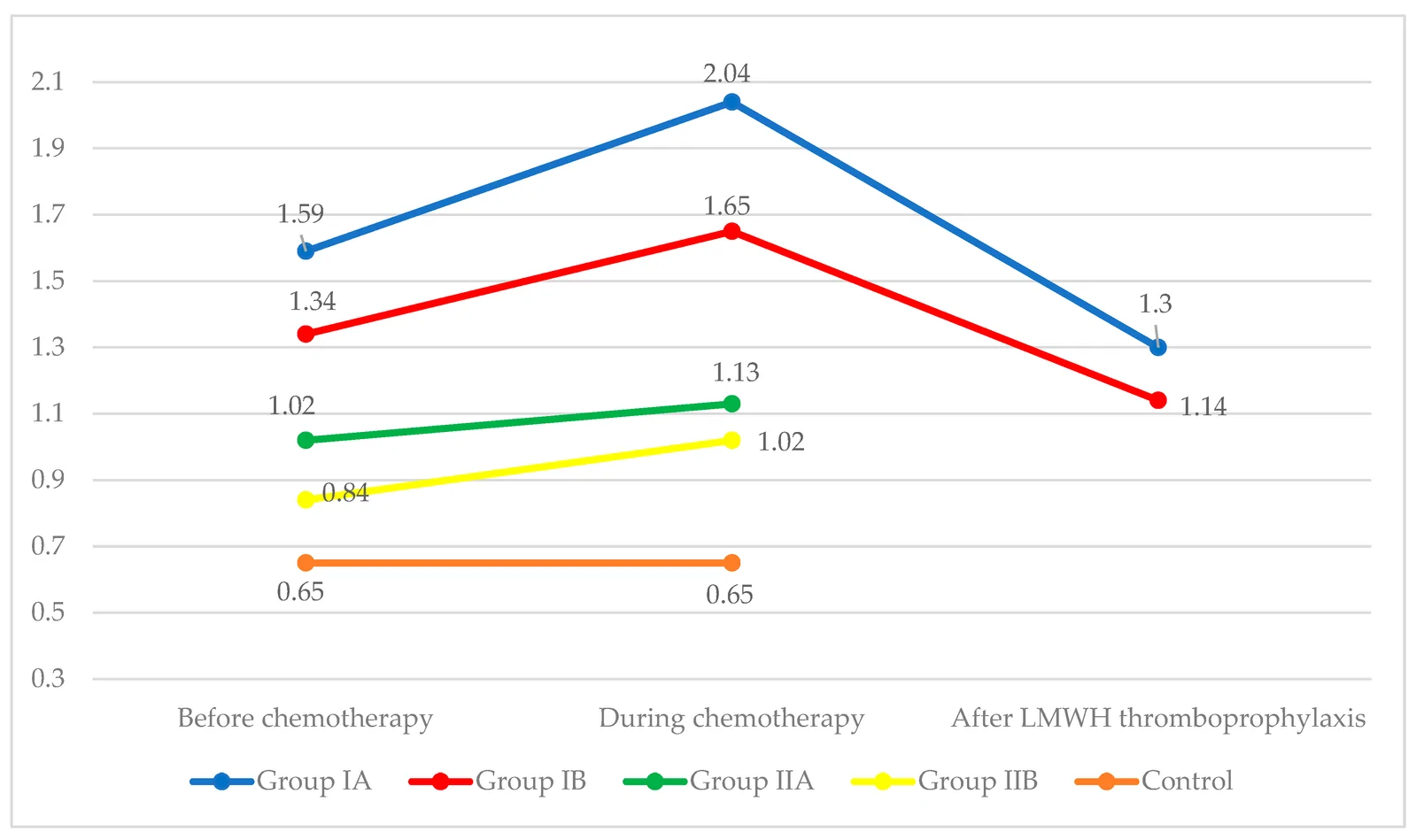
Serial changes in the vWF/ADAMTS13 ratio at the pre-chemotherapy assessment, after 1–2 chemotherapy cycles, and following the chemotherapy/LMWH treatment interval in Group I. No corresponding third time-point assessment was available for Group II or the control group.

The present study demonstrates a pronounced imbalance in the vWF-ADAMTS13 axis in patients with malignancies of the female reproductive system, with the greatest abnormalities observed in those with a history of thrombotic complications. This imbalance was characterized by elevated vWF levels, reduced ADAMTS13 antigen levels and functional activity, and increased concentrations of the ADAMTS13 inhibitor. Consequently, the vWF/ADAMTS13 ratio increased progressively from the control group to patients without previous VTE and reached its highest values in Group I, particularly in subgroup IA. After 1–2 cycles of chemotherapy, the axis showed a further shift toward endothelial–hemostatic imbalance. In Group I, the subsequent assessment following chemotherapy and LMWH thromboprophylaxis demonstrated changes in the opposite direction, with a reduction in the vWF/ADAMTS13 ratio. Thus, serial assessment of the vWF/ADAMTS13 ratio consistently reflected changes in endothelial–hemostatic balance across the evaluated treatment periods (Figure 1).

These findings should be considered in the context of the contemporary concept of thromboinflammation. Cancer-associated thrombosis results not only from activation of the coagulation cascade but also from complex interactions among tumor cells, monocytes and neutrophils, platelets, and the vascular endothelium. Tissue factor, proinflammatory cytokines, tumor-derived extracellular vesicles, and neutrophil extracellular traps contribute to endothelial and hemostatic activation, while activated endothelial cells, in turn, acquire proinflammatory and prothrombotic properties. This creates a self-sustaining inflammation–endotheliopathy–coagulation loop that plays an important role in the pathogenesis of cancer-associated thrombosis [8,9]. Endothelial dysfunction in malignancy is accompanied by increased expression of adhesion molecules, reduced antithrombotic properties of the endothelium, and enhanced interactions with platelets and leukocytes.

The vWF-ADAMTS13 axis occupies a central position within this system. vWF is synthesized predominantly by endothelial cells and released from Weibel–Palade bodies upon endothelial activation. In the circulation, the most thrombogenic ultra-large vWF multimers promote platelet capture, adhesion, and aggregation. Their activity is physiologically controlled by the metalloprotease ADAMTS13, which cleaves high-molecular-weight vWF multimers. Therefore, excessive vWF release combined with a relative reduction in ADAMTS13 activity creates conditions favoring a persistent prothrombotic state [5]. Direct morphological evidence supporting this mechanism in tumor tissue was provided by Karampinis et al., who identified intravascular vWF fibers formed upon endothelial activation and capable of promoting platelet binding and aggregation [4]. These structures were more prominent in patients with VTE and were associated with an unfavorable prognosis.

Of particular interest is not merely an increase in vWF or a decrease in ADAMTS13 considered separately, but rather the imbalance between these two components. Elevated vWF reflects the extent of endothelial activation but does not characterize the capacity of the system to restrain its prothrombotic activity. Conversely, reduced ADAMTS13 becomes particularly relevant in the presence of an excess of its substrate. Thus, the vWF/ADAMTS13 ratio integrates two interrelated processes: the release of prothrombotic vWF and insufficient proteolytic regulation of its activity.

This concept is consistent with the findings of the Vienna Cancer and Thrombosis Study, which included 795 patients with cancer. High vWF levels were independently associated with the subsequent risk of VTE, whereas ADAMTS13 activity alone showed no such association. In contrast, a low ADAMTS13/vWF ratio was associated with a markedly increased risk of VTE: the 2-year cumulative incidence of thrombosis was 10.7% among patients with values below the 25th percentile compared with 2.6% among those with values above the 75th percentile [3]. These findings are particularly relevant to the interpretation of our results because they indicate that the clinically meaningful feature is the imbalance between the two components of the axis.

In the study by Karampinis et al., increased vWF was accompanied by reduced ADAMTS13 activity and an increased vWF/ADAMTS13 ratio. A high ratio was associated with both VTE development and poorer overall survival. Moreover, an increased vWF/ADAMTS13 ratio was already detectable in localized and even in situ malignancies, supporting the possibility that this imbalance may develop early during carcinogenesis [4].

Our findings are consistent with these observations. Even before the initiation of chemotherapy, the vWF/ADAMTS13 ratio was 1.02 in subgroup IIA and 0.84 in subgroup IIB, compared with 0.65 in the control group, indicating that disruption of this axis was already present in patients without previously documented VTE. In patients with a history of thrombosis, the imbalance was substantially more pronounced, reaching 1.59 in subgroup IA and 1.34 in subgroup IB. Taken together, these findings support the interpretation of the vWF/ADAMTS13 ratio as an integrative marker of endothelial–hemostatic imbalance rather than as a measure determined solely by a history of VTE.

A particularly important finding of the present study was the further increase in the vWF/ADAMTS13 ratio after 1–2 cycles of chemotherapy. This pattern was observed across all oncologic subgroups and was most pronounced in Group I. The ratio increased from 1.59 to 2.04 in subgroup IA and from 1.34 to 1.65 in subgroup IB, while changes in the same direction were also observed in subgroups IIA and IIB. Thus, the post-chemotherapy assessment showed a further shift in the vWF-ADAMTS13 axis toward a prothrombotic state compared with the pre-chemotherapy assessment.

This observation has a plausible biological basis. Chemotherapeutic agents can directly injure the vascular endothelium, induce oxidative stress, reduce nitric oxide bioavailability, disrupt endothelial barrier integrity, and increase the expression of proinflammatory and prothrombotic molecules [10]. Platinum-based chemotherapy has been associated with both acute impairment of endothelial function and increased circulating vWF levels after treatment [11]. In a clinical study of patients receiving cisplatin, endothelial dysfunction developed early during treatment, supporting the possibility of a rapid vascular response to cytotoxic therapy.

From this perspective, it is particularly relevant that the post-chemotherapy changes in our study involved not a single biomarker but the entire axis: increased vWF was accompanied by decreased ADAMTS13 antigen levels and functional activity and increased ADAMTS13 inhibitor concentrations. This combined pattern reflects more than an isolated increase in an acute-phase protein; rather, it indicates a shift in the balance between endothelial vWF release and its physiological regulation. Prospective data from other investigators also support an association between changes in the vWF-ADAMTS13 axis and thrombosis during chemotherapy. In the study by Setiawan et al., patients who developed deep vein thrombosis during three months of chemotherapy had higher vWF levels and lower ADAMTS13 levels. The vWF/ADAMTS13 ratio was also significantly higher in patients who subsequently developed DVT, and its increase over time was more pronounced than in patients without thrombosis [12]. These findings are consistent with our results and support the potential use of serial vWF/ADAMTS13 measurements as an indicator of an increasing endothelial–prothrombotic imbalance during anticancer treatment.

Another important observation was the partial reversal of the preceding abnormalities at the assessment performed following chemotherapy and LMWH thromboprophylaxis in Group I. In subgroups IA and IB, ADAMTS13 antigen levels increased, ADAMTS13 inhibitor and vWF concentrations decreased, and the vWF/ADAMTS13 ratio declined from 2.04 to 1.30 and from 1.65 to 1.14, respectively. ADAMTS13 functional activity also increased, although these changes did not reach statistical significance. Because LMWH was administered on clinical grounds only to patients in Group I and no corresponding time-matched third assessment was performed in Group II, these changes cannot be attributed specifically to nadroparin. Nevertheless, their direction is consistent with the hypothesis that anticoagulant treatment may be accompanied by attenuation of endothelial–thromboinflammatory activation, a possibility that warrants evaluation in appropriately controlled prospective studies.

This interpretation is consistent with evidence regarding the pleiotropic properties of heparins. In addition to their anticoagulant activity through potentiation of antithrombin and inhibition of factors Xa and IIa, LMWHs have been reported to exert anti-inflammatory effects through interactions with cytokines and chemokines, complement components, cell adhesion molecules, and selectins [13,14]. Contemporary reviews have also considered LMWHs as compounds capable of modulating cell adhesion, inflammatory signaling pathways, and endothelial function.

The endothelial glycocalyx deserves particular attention in this context. Damage to the glycocalyx increases vascular permeability, exposes adhesion molecules, and facilitates leukocyte and platelet interactions with the endothelial surface. In an experimental model, Lipowsky and Lescanic demonstrated that LMWH dose-dependently reduced inflammation-induced shedding of endothelial glycocalyx components, presumably through inhibition of heparanase and stabilization of the endothelial surface layer [15]. This mechanism could potentially contribute to attenuation of endotheliopathy and thromboinflammation, although its clinical relevance in patients with cancer requires further investigation.

Another potential mechanism involves attenuation of thrombin-dependent inflammatory activation. Thrombin is involved not only in fibrin formation but also in cellular signaling through protease-activated receptors (PARs), thereby promoting activation of endothelial cells, platelets, and inflammatory cells. Consequently, suppression of thrombin generation by LMWH could potentially attenuate the positive feedback loop between coagulation and inflammation. From this perspective, the anticoagulant and anti-inflammatory effects of LMWH should not necessarily be regarded as independent phenomena, but rather as different components of its influence on a unified thromboinflammatory process [16].

However, our findings do not establish a direct endothelium-protective effect of nadroparin. Much of the evidence regarding the non-anticoagulant effects of LMWH derives from experimental studies, and the magnitude of these effects may depend on molecular weight, heparin structure, and the specific biological mechanism involved. Therefore, the reduction in the vWF/ADAMTS13 ratio observed during the chemotherapy/LMWH treatment interval should be interpreted as a treatment-associated longitudinal change rather than as evidence of a specific effect or molecular mechanism of nadroparin.

Nevertheless, the dynamic behavior of the vWF/ADAMTS13 ratio represents one of the most important findings of the present study. The ratio increased at the post-chemotherapy assessment and subsequently decreased during the chemotherapy/LMWH treatment interval in Group I. These serial changes support further investigation of the vWF/ADAMTS13 ratio as a dynamic indicator of endothelial–hemostatic imbalance during anticancer treatment.

## 5. Limitations

This study has several limitations. First, the pre-chemotherapy assessment was performed after surgical treatment and therefore does not represent a preoperative or treatment-naive baseline. Although sampling was undertaken in the late postoperative period after clinical recovery and 1–3 days before the initiation of chemotherapy, a residual effect of surgery on vWF and ADAMTS13 cannot be completely excluded. The timing of sampling nevertheless reduces the likelihood that the observed abnormalities predominantly reflected the postoperative response. Second, LMWH thromboprophylaxis was clinically indicated only in Group I, and no corresponding time-matched third assessment was performed in Group II. Consequently, the changes observed during the chemotherapy/LMWH treatment interval cannot be attributed specifically to nadroparin, and contributions from the natural time course, chemotherapy-cycle timing, or regression to the mean cannot be excluded. Third, the present study represents an extended mechanistic analysis of the same patient cohort reported in our previous study [7] and was designed to characterize serial changes in the vWF–ADAMTS13 axis rather than to validate prediction of incident VTE. Accordingly, the present findings should be interpreted as cross-sectional and treatment-associated biomarker differences rather than as evidence of prognostic performance. Finally, the relatively small subgroup sizes and heterogeneity of tumor type and chemotherapy exposure may limit the generalizability of the findings. Independent prospective studies with standardized sampling schedules and appropriate time-matched comparison groups are required to validate the vWF/ADAMTS13 ratio as a dynamic marker of endothelial–hemostatic imbalance.

## 6. Conclusions

In patients with malignancies of the female reproductive system, an imbalance in the vWF-ADAMTS13 axis was identified, characterized by increased vWF levels, reduced ADAMTS13 antigen levels and functional activity, and increased ADAMTS13 inhibitor concentrations. The most pronounced alterations were observed in patients with a history of thrombotic complications; however, disruption of this axis was also detected in patients without previous VTE. The post-chemotherapy assessment demonstrated a further shift toward endothelial–hemostatic imbalance, whereas the subsequent assessment following chemotherapy and LMWH thromboprophylaxis in Group I showed partial reversal of these changes. The vWF/ADAMTS13 ratio most consistently reflected the direction of the observed changes and may therefore represent a potential integrative marker for dynamic assessment of endothelial–hemostatic imbalance during anticancer treatment. Further prospective studies with time-matched comparison groups are required to determine its clinical utility.

## Figures and Tables

**Table 1:** Baseline parameters of the vWF–ADAMTS13 axis after surgery and before chemotherapy.

Parameter	Group I		Group II		Control Group (n = 25)
	Ovarian Cancer (n = 23)	Cervical Cancer (n = 11)	Ovarian Cancer (n = 20)	Cervical Cancer (n = 20)	
ADAMTS13 level, IU/L (reference range 400–1400)	1188 (803–1650) *^#^	1317 (1012–1723) *^#^	1402 (1201–1824) ^#^	1511 (1257–2032)	1572 (1397–2239)
ADAMTS13 activity (reference range 0.4–1.3)	0.815 ± 0.256 ^#^	0.992 ± 0.187 ^#^	0.908 ± 0.179 ^#^	1.126 ± 0.185	1.256 ± 0.137
ADAMTS13 inhibitor, IU/mL (reference range <0.4)	6.35 ± 2.76 *^#^	4.91 ± 1.78 *^#^	2.14 ± 0.63 ^#^	1.62 ± 0.78 ^#^	0.25 ± 0.17
vWF level, IU/L (reference range 500–1500)	1892 (1239–2369) *^#^	1763 (1013–2092) *^#^	1428 (902–1681) ^#^	1272 (1087–1648) ^#^	1014 (726–1486)
vWF/ADAMTS13 ratio	1.59 *^#^	1.34 *^#^	1.02 ^#^	0.84 ^#^	0.65

* indicates a statistically significant difference (*p* < 0.05) between the corresponding subgroups of Groups I and II; ^#^ indicates a statistically significant difference (*p* < 0.05) between the respective study subgroup and the control group (Table 3).

**Table 2:** Parameters of the vWF–ADAMTS13 axis after 1–2 cycles of chemotherapy.

Parameter	Group I		Group II		Control Group (n = 25)
	Ovarian Cancer (n = 23)	Cervical Cancer (n = 11)	Ovarian Cancer (n = 20)	Cervical Cancer (n = 20)	
ADAMTS13 level, IU/L (reference range 400–1400)	943 (756–1532) *^#^	1084 (813–1627) *^#^	1337 (1154–1761) ^#^	1371 (1265–1803) ^#^	1572 (1397–2239)
ADAMTS13 activity (reference range 0.4–1.3)	0.711 ± 0.352 ^#^	0.773 ± 0.310 *^#^	0.876 ± 0.204 ^#^	1.106 ± 0.197 ^#^	1.256 ± 0.137
ADAMTS13 inhibitor, IU/mL (reference range <0.4)	7.58 ± 3.52 *^#^	5.18 ± 2.71 *^#^	2.64 ± 1.04 *^#^	2.13 ± 0.89 *^#^	0.25 ± 0.17
vWF level, IU/L (reference range 500–1500)	1923 (1321–2517) *^#^	1791 (1178–2259) *^#^	1513 (1126–2141) *^#^	1396 (1027–2064) *^#^	1014 (726–1486)
vWF/ADAMTS13 ratio	2.04 *^#^	1.65 *^#^	1.13 *^#^	1.02 *^#^	0.65

* indicates a statistically significant difference (*p* < 0.05) between the corresponding subgroups of Groups I and II; ^#^ indicates a statistically significant difference (*p* < 0.05) between the respective study subgroup and the control group (Table 4).

**Table 3:** Statistical comparisons of baseline parameters of the vWF–ADAMTS13 axis.

Parameter	*p*-Values					
	IA vs. IIA	IB vs. IIB	IA vs. Control	IB vs. Control	IIA vs. Control	IIB vs. Control
ADAMTS13 level, IU/L (reference range 400–1400)	0.008	0.031	0.002	0.018	0.039	0.341
ADAMTS13 activity (reference range 0.4–1.3)	0.171	0.069	0.002	0.006	0.003	0.063
ADAMTS13 inhibitor, IU/mL (reference range <0.4)	0.001	0.003	0.001	0.001	0.001	0.002
vWF level, IU/L (reference range 500–1500)	0.003	0.011	0.001	0.002	0.006	0.018
vWF/ADAMTS13 ratio	0.006	0.009	0.001	0.002	0.012	0.027

**Table 4:** Statistical comparisons of the vWF–ADAMTS13 axis parameters after 1–2 cycles of chemotherapy.

Parameter	*p*-Values					
	IA vs. IIA	IB vs. IIB	IA vs. Control	IB vs. Control	IIA vs. Control	IIB vs. Control
ADAMTS13 level, IU/L (reference range 400–1400)	0.004	0.018	0.001	0.003	0.012	0.021
ADAMTS13 activity (reference range 0.4–1.3)	0.064	0.012	0.001	0.003	0.002	0.014
ADAMTS13 inhibitor, IU/mL (reference range <0.4)	0.001	0.004	0.001	0.001	0.001	0.002
vWF level, IU/L (reference range 500–1500)	0.006	0.021	0.001	0.002	0.007	0.016
vWF/ADAMTS13 ratio	0.006	0.021	0.001	0.003	0.009	0.014

**Table 5:** Longitudinal changes in laboratory parameters in Group I before and after the chemotherapy/LMWH treatment interval.

Parameter	Before LMWH: Ovarian Cancer (n = 23)	Before LMWH: Cervical Cancer (n = 11)	After LMWH: Ovarian Cancer (n = 23)	After LMWH: Cervical Cancer (n = 11)	*p*-Value	
					IA: Before vs. After LMWH	IB: Before vs. After LMWH
ADAMTS13 level, IU/L (reference range 400–1400)	943 (756–1532) *	1084 (813–1627) *	1325 (1278–1641)	1426 (1212–1842)	0.004	0.007
ADAMTS13 activity (reference range 0.4–1.3)	0.711 ± 0.352	0.773 ± 0.313	0.843 ± 0.121	0.952 ± 0.163	0.100	0.120
ADAMTS13 inhibitor, IU/mL (reference range <0.4)	7.58 ± 3.52 *	5.18 ± 2.71 *	1.73 ± 0.57	1.48 ± 0.51	0.003	0.006
vWF level, IU/L (reference range 500–1500)	1923 (1321–2517) *	1791 (1178–2259) *	1684 (1252–1845)	1567 (1234–1623)	0.012	0.038
vWF/ADAMTS13 ratio	2.04 *	1.65 *	1.30	1.14	0.004	0.009
D-dimer (reference range <550 ng/mL)	2783 ± 953 *	2613 ± 1021*	512 ± 73	497 ± 98	0.001	0.002

* indicates a statistically significant within-group difference between the pre- and post-treatment interval assessments (*p* < 0.05). Corresponding *p*-values are shown in the final two columns.

## Data Availability

The raw data supporting the conclusions of this article will be made available by the authors on request.

## Supplementary Materials

The following supporting information can be downloaded at: https://www.mdpi.com/article/10.3390/biomedicines14092078/s1, Table S1. Baseline clinical characteristics and planned chemotherapy exposure of the study cohort.
