# Supplementary material for: The vWF/ADAMTS13 Ratio as a Potential Marker of Endotheliopathy in Malignancies
Source: Biomedicines. 2026 Sep 15;14(9):2078. doi: 10.3390/biomedicines14092078 (PMC13604419; doi:10.3390/biomedicines14092078)
Supplement: Supplementary file 1 [file biomedicines-14-02078-s001.zip › biomedicines-4549055-supplementary.pdf]

Supplementary Materials

Table S1. Baseline clinical characteristics and planned chemotherapy exposure of the study cohort.

Characteristic	Group I		Group II	
	**Ovarian Cancer (n = 23)**	**Cervical Cancer (n = 11)**	**Ovarian Cancer (n = 20)**	**Cervical Cancer (n = 20)**
Age 30–35 years, n (%)	2 (8.7)	1 (9.1)	2 (10.0)	1 (5.0)
Age 40–45 years, n (%)	4 (17.4)	3 (27.3)	3 (15.0)	5 (25.0)
Age 45–50 years, n (%)	9 (39.1)	4 (36.4)	8 (40.0)	8 (40.0)
Age 50–55 years, n (%)	6 (26.1)	2 (18.2)	5 (25.0)	4 (20.0)
Age 55–60 years, n (%)	2 (8.7)	1 (9.1)	2 (10.0)	2 (10.0)
Stage I, n (%)	5 (21.7)	2 (18.2)	4 (20.0)	5 (25.0)
Stage II, n (%)	7 (30.4)	4 (36.4)	7 (35.0)	7 (35.0)
Stage III, n (%)	11 (47.8)	5 (45.5)	9 (45.0)	8 (40.0)
Planned 4 chemotherapy cycles, n (%)	4 (17.4)	1 (9.1)	3 (15.0)	1 (5.0)
Planned 6 chemotherapy cycles, n (%)	5 (21.7)	8 (72.7)	4 (20.0)	16 (80.0)
Planned 8 chemotherapy cycles, n (%)	14 (60.9)	2 (18.2)	13 (65.0)	3 (15.0)
